# Elastic network model of allosteric regulation in protein kinase PDK1

**DOI:** 10.1186/1472-6807-10-11

**Published:** 2010-05-25

**Authors:** Gareth Williams

**Affiliations:** 1 Wolfson Centre for Age-Related Diseases, Kings College London, London Bridge, London SE1 1UL, UK

## Abstract

**Background:**

Structural switches upon binding of phosphorylated moieties underpin many signalling networks. The ligand activation is a form of allosteric modulation of the protein, where the binding site is remote from the structural change in the protein. Recently this structural switch has been elegantly demonstrated with the crystallisation of the activated form of 3-phosphoinositide-dependent protein kinase-1 (PDK1). The purpose of the present work is to determine whether the allosteric coupling in PDK1 emerges at the level of a simple coarse grained model of protein dynamics.

**Results:**

It is shown here that the allosteric effects of the agonist binding to the small lobe upon the activation loop in the large lobe of PDK1 are explainable within a simple 'ball and spring' elastic network model (ENM) of protein dynamics. In particular, the model shows that the bound phospho peptide mimetic fluctuations have a high degree of correlation with the activation loop of PDK1.

**Conclusions:**

The ENM approach to small molecule activation of proteins may offer a first pass predictive methodology where affinity is encoded in residues remote from the active site, and aid in the design of specific protein agonists that enhance the allosteric coupling and antagonist that repress it.

## Background

Phosphorylation dependent protein interactions are a common feature of biological processes [1]. A relevant kinase attaches a phosphate group to a specific tyrosine, threonine or serine residue on the protein surface resulting in a considerable increase in the binding affinity for a target protein. The binding event triggers a conformational change whereby the target protein switches from inactive to active state or vice versa [2]. It is not possible to generalise the nature of the conformational change and it is only with crystallisation that light has been thrown on the mechanism. PDK1 in common with other AGC kinases have a catalytic domain consisting of an N-terminal small lobe harbouring a PDK1-interacting fragment (PIF) binding pocket, a large C-terminal lobe with an activation loop and an ATP binding site in the cleft between the two lobes [3]. The PDK1 kinase is activated by a phosphorylated peptide binding to the PIF pocket [4]. This binding triggers not only local conformational changes in the pocket and the ATP binding site, but also in the remote activation loop. Structural insight into the mechanism of activation of PDK1 has been gained by the crystallisation in the first instance of the inactive version of the protein [5] where the activation loop appears unstructured. And the recent development of the first small molecule protein kinase agonist targeting the PIF binding pocket in PDK1 has enabled the crystallisation of the active form of PDK1 and here the activation loop is ordered [6]. Remarkably, the binding of the small ligand induces a significant and critical structural change at a remote site on the protein. The present study addresses the question as to whether these changes could have been predicted (and/or can be explained) by molecular modelling.

The elastic network model (ENM) or Gaussian network model is a simple 'ball and spring' model introduced to describe full atomic fluctuations within the protein [7] and then to model the deformations of the C*α *backbone [8,9]. The initial successful application of the model showed remarkable agreement between the scalar expectation value of residue fluctuations with crystallographic B-factors or temperature factors [8,10]. Over the past few years an ENM of protein dynamics has emerged as a viable theoretical framework for the study of allosteric regulation in protein signalling. An ENM model of the chaperonin GroEL has revealed a dominant eigenvector describing the allosteric switch in the protein and first order perturbation theory predicts the critical residues in this transition [11], Recently, Balabin *et al *[12] have looked at the coupling of local fluctuations within GPCR. They show that the distinct features of rhodopsin and *β*2-adrenoreceptor activation are encoded in the correlation between these fluctuations and that there are clear allosteric couplings between ligand binding sites outside the membrane and G-protein binding sites within the cell. Protein functional sites that result in structural changes in the protein have also been successfully described using a dynamics perturbation analysis (DPA) [13-16]. Here an interacting ENM comprising the protein and a series of probes covering the protein surface is set up and those probes that couple to the biggest changes in protein conformational distribution are predicted to lie at interaction sites. The present study applies a version ENM to the protein kinase PDK1 and it is demonstrated that the allosteric coupling between the PIF binding site and the activation loop is encoded in the fluctuation correlation coefficient of the vibrational excitations of the protein.

## Methods

### Elastic Network Model

In the ENM the protein is approximated by the C*α *atoms vibrating about the equilibrium crystal coordinates coupled through a quadratic potential. Explicitly the potential takes the form(1)

where  is the distance between residue C*α *coordinates *x_i_^*μ*^*, with *i *= 1, ..., L and L is the protein length, and Greek letters referring to spatial degrees of freedom.  are the equilibrium distances observed in the crystal structure, is a radius of influence ~15Å and *γ*_*ij *_is the spring constant. The spring constant will be taken to depend only on the residue type *γ*_*ij *_= *δ*(*s*_*i*_, *a*)*δ*(*s*_*j*_, *b*)*γ*_*ab*_, where *S_i _*is the protein sequence and *a b *= 1,..., 20. Expanding the potential to second order in fluctuations about the equilibrium conformation we get(2)

where , and the 3L × 3L Hessian matrix is given by(3)

and(4)

where the 'hat' indicates a unit vector. The eigenvalues of the Hessian matrix correspond to the protein vibrational normal modes. There are six zero modes corresponding to the rotational and translational symmetries, low frequency modes that couple remote parts of the protein and high frequency components that are residue autonomous [17]. The diagonalisation in this study was performed by first reducing the matrix to a tridiagonal form with the Housholder algorithm and then employing the QL algorithm, see [18].

Within the harmonic oscillator model the coupling of residue fluctuations is defined as the expectation value , where  is the partition function and the integral is over all conformations, is the temperature and *k_B _*the Boltzmann constant. Expanding the potential in powers of the fluctuation Eq. 1, introducing a source term *J *and defining , where *Dr *is the integral over all configurations of *r*, it follows that  and therefore(5)

where *λ*_*n *_are the eigenvalues and  the eigenvectors of the Hessian matrix Eq. 2, , see [17]. Only fluctuations that break the rotational/translational symmetry are considered and therefore the zero modes are dropped. Note that in contrast to isotropic ENM Kirchhoff matrix treatment the Hessian matrix is not diagonal in the spatial degrees of freedom and the spring constant isn't an overall factor in the correlation. Hence, the usual factor  is missing from Eq. 5.

As it stands the correlation Eq. 4 is a tensor and a general contraction can be got through an arbitrary rotation matrix, *R_*μν*_*, so that the scalar correlation is now(6)

where *Tr *is the trace. In the present study the rotation matrix is taken to be the identity matrix. It can be shown that , where *σ*^*μ *^are the principle components of *C *and these can be got through singular value decomposition [19]. A natural choice would then be . However, the results were not fundamentally altered with this additional step and it is therefore not presented. The correlation matrix has to be normalised relative to the self correlation [17] so that finally(7)

Now extending this analysis to the case of ligand interactions, an allosteric effect of the ligand binding event upon the protein will show up as a peak in the correlation function Eq. 7 between the two sets of network nodes. Given ligand coordinates , *i *= 1, ..., *N'*, the positive definite allosteric profile is here defined as(8)

### Spring constant

The amino acid type determines the interaction strength, for simplicity this can be encoded as a residue pair dependent spring constant *γ*_*ab*_, where the indices refer to amino acid type. The extent of a residue's fluctuation is measured by the B-factor in the pdb file. The B-factor is given by  and the energy for an oscillating pair is so that the spring constant goes like the inverse of the B-factor and as a first approximation(9)

The average B-factor for each amino acid is obtained by scanning the PDBselect25 database of non-redundant pdb files [20]. The values for the residue specific B-factors are given in Additional file 1. An alternative definition of the residue specific interaction energy can be defined based on residue contact frequencies in a database of protein structures. Here, the energy is given by , where *n_ab _*(*R_c_*) is the number of non-chain proximal residue pairs, type *a *and *b*, within an interaction radius and *N_a _*is the number of residues of a given type in the proteins, this is essentially the MJ matrix [21,22]. The two energy measures correlate very well, with *Rc *= 15Å the Pearson correlation is *r *= 0.89.

## Results and Discussion

**Figure 1:**
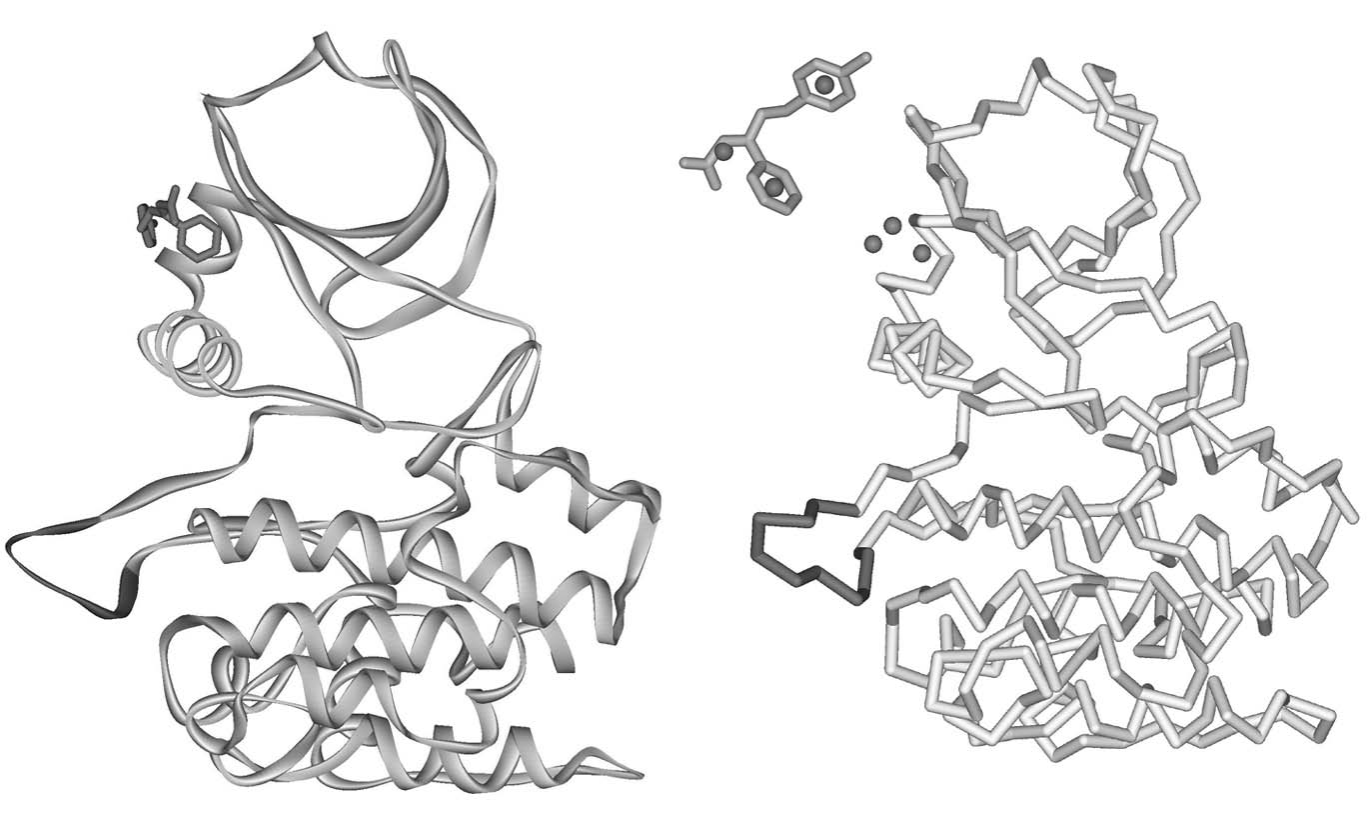
**The crystal structure of the protein kinase PDK1**. The protein is shown as a solid ribbon and the agonist PS48 as a stick diagram, protein data bank accession 3HRF. The activation loop is highlighted in black. To the right is the same protein fold as a C*α *ENM, with the agonist replaced by three nodes. The node positions relative to PS48 are shown above.

The present study addresses the allosteric coupling between a ligand agonist bound to the phospho-peptide binding site, the PIF pocket, and the activation loop of protein kinase PDK1. The structure of the activated protein has been solved and has the protein data bank accession code 3HRF [6]. In figure 1 the protein is shown as a ribbon diagram with the phospho-peptide mimetic PS48 and the activation loop in dark grey. Comparing this structure with the free PDK1 (1H1W) [5] it is clear that the activation loop in the C-terminal domain undergoes a conformational change upon PS48 binding to the N-terminal domain, see below.

**Figure 2:**
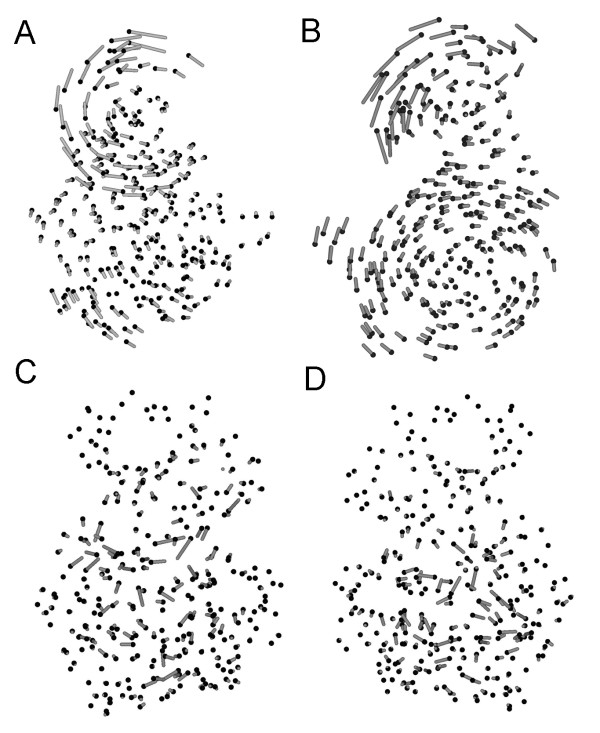
**The low and high energy mode distortions of PDK1**. The distortions due to the first two non-zero mode eigenvectors are shown at the top. The black spheres depict the C*α *crystal coordinates and the grey lines correspond to the normal mode displacement of these. The motion consists of a counter twisting of the small and large lobes of the protein kinase PDK1. In contrast, high energy modes of excitation are characterised by local fluctuations and low coherence. Modes 852 and 850 are illustrated below.

Low energy non-zero modes contribute to global protein vibrational excitations, the first six modes are zero modes corresponding to the three translational and three rotational degrees of freedom. In figures 2A and 2B the structural distortion effected by the first two non-zero modes are illustrated. The small and large lobes of PS48 are clearly depicted and a major component of these modes is a counter twisting of the two lobes. This is an intuitively obvious major contribution to the internal energy of a binary object. In contrast, the high energy part of the spectrum, figures 2C and 2D, is characterised by local fluctuations and the lack of coherence across the protein. The vibrational spectrum is dependent on the influence radius, see methods Eq. 1, and this is chosen so that the self correlation *C_ii_*, Eq. 5, has maximal correlation with the crystallographic B-factors [8] so thatÅ. However, it will be shown that the results below are robust against variation of the model parameters.

In the present case the molecule PS48 consists of 20 heavy atoms and has three distinct moieties comprising two phenyl rings and a carboxylate. It is reasonable to replace this molecule with three centroids as illustrated in figure 1 and this is done by K-means clustering [23]. The spring constant, Eq. 1, for these centroids coupling to the protein backbone C*α*'s and to themselves is taken to be the average of the inter-amino acid spring constant, , where the tick refers to small molecule centroid.

**Figure 3:**
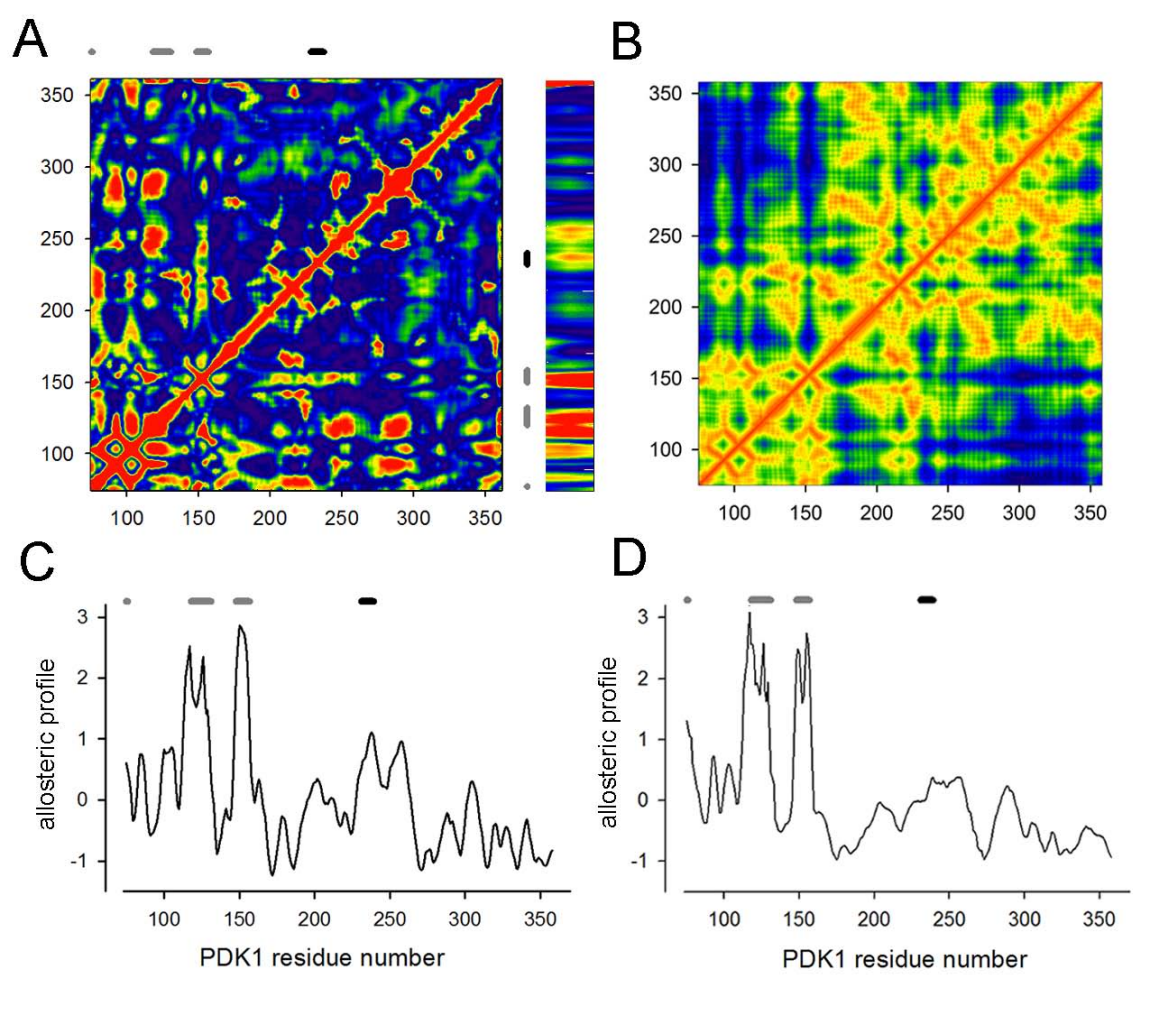
**The allosteric profile and fluctuation correlation plots for PDK1**. The correlation matrix shown as an intensity plot A, with the direct agonist contacts shown as gray bars and the activation loop as a black bar. The agonist part of the correlation matrix is expanded and shown to the right of A for clarity. There is a clear peak in the correlation at the activation loop. The distance matrix is shown in B and the allosteric sites in A are those peaks that are away from direct residue contacts. A linear allosteric profile for PS48 across the protein is shown in C, with the direct ligand contacts shown as grey bars and the activation loop as a black bar above the graph. The highest correlation in the large lobe is at the activation loop. When the agonist is removed from the crystal and the correlation of the would be agonist interacting residues in the PIF pocket with the rest of the protein is calculated the peak shifts to residue THR255 away from the activation loop, D. Note, the correlation profiles have been scaled to average zero and have a standard deviation of unity.

The fluctuation correlation matrix Eq. 7 is shown in figure 3A. The PDK1 residues in direct contact with the agonist are highlighted with grey bars and not surprisingly correspond to correlation peaks. The activation loop position is highlighted with a black bar and it is clear that the agonist has a high degree of allosteric coupling to this loop. The distance matrix, , for PDK1 is shown in figure 3B and this shows that proximal residues correspond to peaks in the correlation matrix. The allosteric correlations are by definition away from distance matrix minima. The allosteric profile Eq. 8 of PS48 against the protein is given in figure 3C. The profile shows a peak in the large lobe at residue ARG238, which is within the activation loop, residues 230-240 highlighted in figure 1. The small lobe peaks correspond to direct ligand protein interactions. The allosteric coupling between the ligand and activation loop is relatively insensitive to the influence radius, with the same result obtaining in the range 10-20Å. The profiles are smoothed over a 5 residue window. It is important to note that the coupling is between the bound ligand and the activation loop and not between the ligand interacting residues and the activation loop. In the absence of the ligand the ligand interaction residues can still be defined as those proximal to the ligand, within an interaction cut-off radius of 5Å the interacting residue number is 14. In figure 3D the coupling between the interaction residues in the PIF pocket and the rest of the protein is shown. Here the picture is somewhat different, with the correlation maximum in the large lobe now at residue THR255, away from the activation loop. The correlation is only slightly higher at residue THR255 than at residue ARG238 and this might reflect the inadequate treatment of the binding site residues or the critical nature of the ligand nodes.

**Figure 4:**
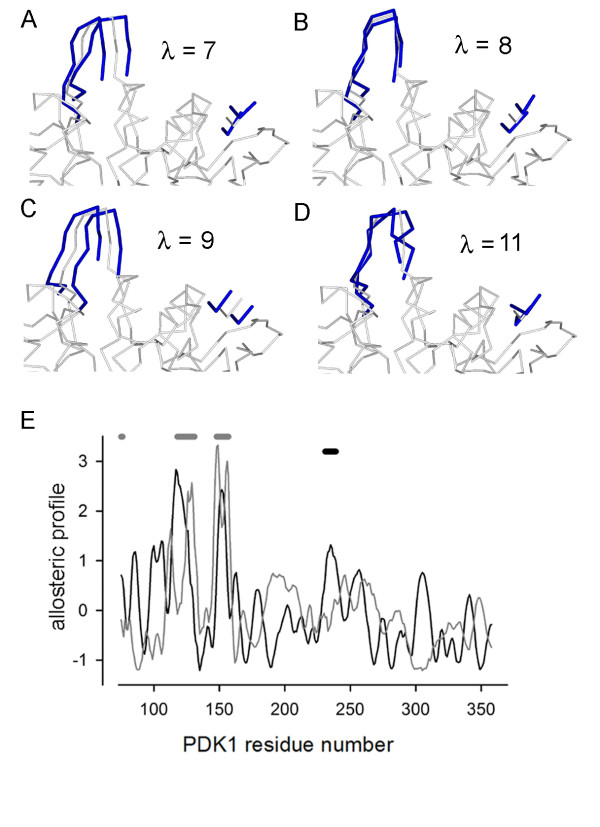
**The allosteric coupling between ligand and activation loop is dominated by four low energy modes**. The allosteric coupling between the PS48 ligand and the activation loop in the large lobe of PDK1 is due to the four low energy modes 7, 8, 9 and 11. The deformations in the activation loop and the ligand are shown in blue in A, B, C and D on top of the crystal backbone structure in grey. The allosteric profile for the coupling modes and the rest of the modes is shown in E. The modes 7, 8, 9 and 11 show a clear dominant peak at ARG238 in the large lobe, black curve. The allosteric profile without these modes is shown in grey and here the coupling is lost.

**Figure 5:**
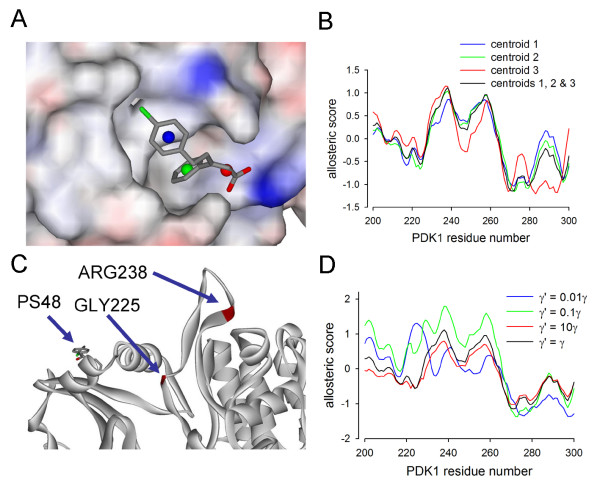
**The coupling of PS48 to the activation loop is distributed over the ligand centroids and depends on the ligand-protein spring constant**. The PS48 agonist ligand has three distinct moieties consisting of two phenyl rings and a carboxylate. These moieties are modelled in the ENM with three centroids coloured blue, green and red, A. When the ligand allosteric profile is calculated with each centroid separately it is apparent that only the middle phenyl ring and carboxylate moieties lead to a dominant correlation at ARG238 in the activation loop, B. The spring constant coupling between the ligand and the protein was taken to be an average over inter amino acid couplings. As the ligand coupling is weakened the allosteric profile undergoes a transition to a new dominant correlation at GLY225, see D, which is buried in the structure, C.

The fluctuation correlation between the ligand and the activation loop is dominated by the low energy modes 7, 8, 9 and 11. The displacements of the ligand and activation loop due to these modes is shown in figures 4A, 4B, 4C and 4D. The allosteric profile for the combination of modes 7, 8, 9 and 11 is shown in black in figure 4E and it is clear that the residue ARG238 peak is dominant in the large lobe. However, when the rest of the modes are summed a different pattern emerges, figure 4E grey curve, with the dominant peak now at residue THR255. In this model the ligand is represented by three centroids that stand for the two phenyl rings and the carboxylate. The allosteric coupling is not evenly distributed over the ligand and this can be seen by setting up the elastic network model with each centroid separately. In figure 5A the ligand is shown buried in the space-filled PDK1 PIF pocket. The centroids are coloured blue, green and red. The allosteric profile for each centroid in turn is shown in figure 5B and it is evident that only centroids 2 (phenyl ring) and 3 (carboxylate) have a dominant activation loop coupling, ARG238 figure 5C. For the mode calculations the coupling spring constant between the ligand centroids and the PDK1 C*α *backbone were set to the average inter-residue spring constants. It is interesting to see the consequences of varying this interaction strength. In figure 5D the allosteric profiles for the ligand across PDK1 is shown. As the spring constant decreases there is a transition to a new allosteric profile with GLY225 now emerging as the dominant peak and this is buried in the structure of PDK1 as shown in figure 5C. Hence, the activation loop coupling is lost for weak ligand-protein interaction strengths.

**Figure 6:**
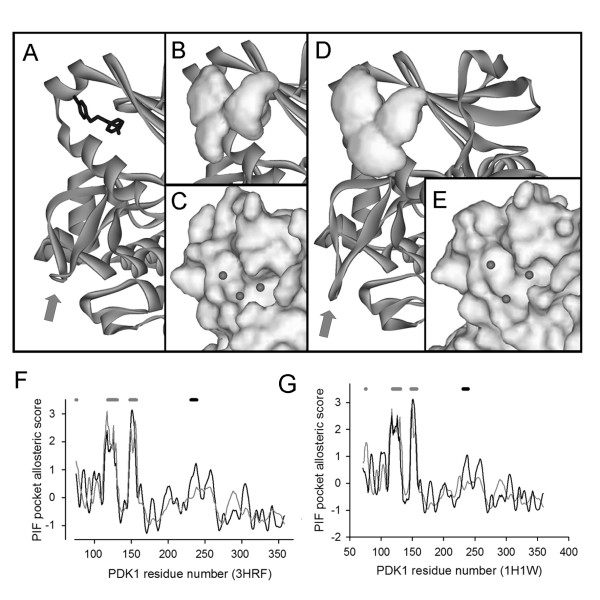
**The allosteric coupling between the PIF pocket and the activation loop is preserved with modelled ligand nodes**. In A the active PDK1 structure is shown as a ribbon diagram with PS48 as a stick structure, the activation loop is picked out with the arrow. The PIF pocket cavity is shown in B and the K-means cluster centres for this cavity are shown in C. The allosteric profile of the PIF pocket nodes is shown in F, with the direct ligand contacts shown as grey bars and the activation loop as a black bar above the graph. There is a clear peak in correlation at the activation loop, as in figure 3. For comparison the allosteric profile for the liagnd interacting residues in the absence of the centroids is shown in grey and here the activation loop is no longer dominant. The inactive PDK1 structure is shown in D, with the four missing residues modelled onto the activation loop, which is picked out with the arrow. The PIF pocket cavity is shown space filled. The K-means clustered nodes are shown in E against the space filled PDK1. The allosteric coupling of the PIF pocket nodes to PDK1 is shown in G and remarkably the activation loop correlation peak is still dominant in the large lobe. In contrast, when the modelled ligand is removed from the network and the allosteric profile for the PIF pocket ligand interacting residues calculated there is no longer a dominant small lobe peak at the activation loop, grey curve in C.

For this analysis to be applied predictively it has to be valid for cases where the ligand-protein complex has not been solved i.e. in the present case for the inactive PDK1 structure. The inactive PDK1 structure still harbours a PIF pocket cavity and this can be used to model a ligand for the ENM analysis. Introducing artificial ligand atoms into an ENM is not new and underlies the DPA methodology for predicting ligand interacting sites [13-16]. In the present case ligand nodes are defined by filling the PIF pocket cavity with 0.5Å spaced points within a Van der Waals radius of the protein heavy atoms and clustering these into a set of three nodes. In the present case a simple K-means clustering was implemented [23]. This approach was first tested on the active PDK1 structure missing PS48 and shown to result in essentially the same allosteric profile, figure 6F. When applied to the inactive PDK1 structure (protein data bank accession 1H1W) the same general picture emerges, see figure 6G. That is, the model predicts the allosteric effects of ligand binding in the absence of structural data on the active PDK1.

## Conclusions

The minimalist 'ball and spring' elastic network model has been applied to the functionally critical allosteric regulation of a protein by ligand binding. The fluctuations induced by the PS48 ligand binding to the PIF pocket in the small lobe of PDK1 are shown to correlate with fluctuations in the remote activation loop in the large lobe of PDK1. The coupling is dominated by the four low energy modes 7, 8, 9 and 11 and is driven by the central phenyl ring and carboxylate moieties of the ligand. The allosteric profile is sensitive to the ligand-protein spring coupling constant and the dominant large lobe peak shifts away from the activation loop to the buried GLY225 when the coupling is weakened. This allosteric coupling is lost when the ligand is removed and substituted by the ligand interacting residues of the protein. However, in a modelled ligand binding structure of the inactive PDK1 protein the PIF-activation loop coupling emerges as the dominant peak in the large lobe allosteric profile. This suggests that the ENM approach can be used to predict the allosteric consequences of modelled agonists.

There are additional sites outside the PIF pocket that undergo structural changes upon ligand binding that are critical for PDK1 activity [6]. These sites, near the ATP binding pocket, are PHE93 in the GLY rich loop GLY89-THR95 and LYS111. The allosteric effects here are in the side chain orientation of PHE93 and LYS111 that leave the local backbone largely unaltered. Within the ENM there does not appear to be any allosteric coupling to the GLY rich loop or to LYS111 and this may be because this model is purely a C*α *reduction of PDK1 and consequently side chain movements are invisible. However, the most stricking difference between agonist bound and unbound structures is in the activation loop, with the unbound PDK1 having unstructured residues GLU233-GLN236 within the activation loop and it is here that the ENM allosteric coupling occurs.

In many instances specificity of protein interaction is encoded by the distinct coupling of conserved active sites, e.g. enzyme catalytic, sites to non-local non-conserved parts of the protein. Also, mutations affecting affinity of catalytic sites or protein interaction sites in general can be sensitive to residue mutations far away from the binding interface. It is clear then that the ENM approach may offer a first pass predictive methodology here. Specifically, ENM can inform mutagenesis experiments, but also aid in the design of specific protein agonists that enhance the allosteric coupling and antagonist that repress it.

## Authors' contributions

GW is the sole author of the present study

## Supplementary Material

Additional file 1 **The ENM residue type specific spring constant**. The residue specific ENM spring constant is derived from the average residue specific crystallographic B-factors across a database of structures. This spring constant is related to a statistical inter-residue energy matrix. Click here for file
