# Supplementary material for: Elastic network model of allosteric regulation in protein kinase PDK1
Source: BMC Struct Biol. 2010 May 25;10:11. doi: 10.1186/1472-6807-10-11 (PMC2888814; doi:10.1186/1472-6807-10-11)
Supplement: Additional file 1 — The ENM residue type specific spring constant. The residue specific ENM spring constant is derived from the average residue specific crystallographic B-factors across a database of structures. This spring constant is related to a statistical inter-residue energy matrix. [file 1472-6807-10-11-S1.DOC]

**Additional file 1**


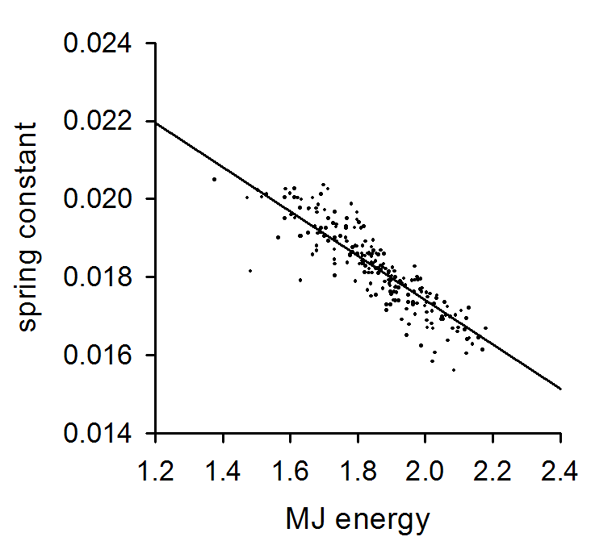


| Residue | B-factor | SEM | B1(n1) | B2(n2) | B3(n3) |
| --- | --- | --- | --- | --- | --- |
| A | 25.911 | 0.039 | 25.960 (4439) | 25.941 (4454) | 25.834 (4376) |
| C | 25.651 | 0.296 | 25.559 (951) | 26.204 (934) | 25.191 (1038) |
| D | 29.937 | 0.303 | 29.601 (3396) | 30.542 (3370) | 29.669 (3344) |
| E | 32.031 | 0.056 | 32.086 (3664) | 31.920 (3943) | 32.088 (3814) |
| F | 24.936 | 0.273 | 24.411 (2388) | 25.328 (2379) | 25.069 (2383) |
| G | 26.974 | 0.318 | 27.354 (4106) | 26.342 (4159) | 27.227 (4031) |
| H | 27.911 | 0.231 | 27.703 (1389) | 27.658 (1359) | 28.373 (1333) |
| I | 24.977 | 0.152 | 24.686 (3524) | 25.044 (3380) | 25.201 (3281) |
| K | 31.138 | 0.141 | 31.417 (3542) | 31.037 (3621) | 30.960 (3586) |
| L | 26.31 | 0.21 | 26.482 (5209) | 26.555 (5184) | 25.892 (5168) |
| M | 27.556 | 0.221 | 27.115 (1256) | 27.758 (1184) | 27.795 (1183) |
| N | 28.806 | 0.226 | 28.723 (2652) | 28.463 (2715) | 29.232 (2618) |
| P | 28.818 | 0.18 | 28.597 (2555) | 28.682 (2601) | 29.175 (2655) |
| Q | 30.278 | 0.585 | 29.399 (2239) | 31.387 (2339) | 30.049 (2183) |
| R | 29.563 | 0.304 | 30.076 (2841) | 29.590 (2875) | 29.024 (2792) |
| S | 28.924 | 0.12 | 28.684 (3480) | 29.060 (3549) | 29.028 (3503) |
| T | 26.872 | 0.249 | 26.473 (3260) | 27.329 (3337) | 26.813 (3282) |
| V | 24.72 | 0.093 | 24.536 (4064) | 24.790 (3966) | 24.833 (4082) |
| W | 24.394 | 0.071 | 24.291 (836) | 24.363 (853) | 24.529 (867) |
| Y | 24.965 | 0.114 | 25.074 (2109) | 24.738 (2058) | 25.083 (2088) |

The average residue specific fluctuation B-factors are given for a database of 1000 proteins from the PDBselect25 database of proteins sharing less than 25% sequence identity. The statistics are generated by randomly assigning each residue in each structure with one of three baskets, shown in the last three columns, and then generating the averages and SEMs. A statistical measure of the residue-residue interaction energy can be obtained from the residue contact frequencies $E_{ab}=-\ln\left(\frac{n_{ab}\left(Rc\right)}{N_{a}N_{b}}\right)$, where $n_{ab}\left(Rc\right)$ is the number of non-chain proximal residue pairs, type *a* and *b*, within an interaction radius and $N_{a}$ is the number of residues of a given type in the proteins this is similar to the Miyazawa-Jernigan matrix, ref 18 in MS. Introducing this into a Lennard-Jones potential model $V_{ab}=-4E_{ab}\left(\left(\frac{\sigma}{r}\right)^{12}-\left(\frac{\sigma}{r}\right)^{6}\right)$ and expanding about the minimum $r_{0}=\sigma2^{\frac{1}{6}}$we get $V_{ab}=E_{ab}-\frac{36E_{ab}}{r_{0}^{2}}\left(r-r_{0}\right)^{2}$. Therefore, if our choice of spring constant is justified it must correlate with the MJ energy and we find a Pearson correlation coefficient of 0.89, top right. The values for the energies are given above the diagonal in the matrix below and the errors below the diagonal. The statistics are generated by splitting the protein database into three databases of ~300 protein chains.

|  |  | A | C | D | E | F | G | H | I | K | L | M | N | P | Q | R | S | T | V | W | Y |
| --- | --- | --- | --- | --- | --- | --- | --- | --- | --- | --- | --- | --- | --- | --- | --- | --- | --- | --- | --- | --- | --- |
|  | A | 1.673 | 1.802 | 1.961 | 1.998 | 1.731 | 1.823 | 1.834 | 1.679 | 2.034 | 1.688 | 1.731 | 1.97 | 1.969 | 1.941 | 1.902 | 1.903 | 1.846 | 1.654 | 1.78 | 1.798 |
| A | 0.014 | C | 0.825 | 1.912 | 2.004 | 1.629 | 1.764 | 1.68 | 1.677 | 1.99 | 1.705 | 1.68 | 1.817 | 1.77 | 1.879 | 1.818 | 1.732 | 1.748 | 1.683 | 1.637 | 1.657 |
| C | 0.016 | 0.04 | D | 2.097 | 2.169 | 1.854 | 2.015 | 1.964 | 1.834 | 2.066 | 1.895 | 1.921 | 2.06 | 2.089 | 2.095 | 2.018 | 2.051 | 2.003 | 1.868 | 1.855 | 1.883 |
| D | 0.011 | 0.009 | 0.025 | E | 2.085 | 1.9 | 2.121 | 2.022 | 1.854 | 2.022 | 1.885 | 1.952 | 2.157 | 2.13 | 2.122 | 1.987 | 2.124 | 2.074 | 1.897 | 1.92 | 1.949 |
| E | 0.021 | 0.01 | 0.025 | 0.01 | F | 1.516 | 1.79 | 1.713 | 1.583 | 1.893 | 1.613 | 1.631 | 1.833 | 1.855 | 1.842 | 1.814 | 1.777 | 1.764 | 1.598 | 1.612 | 1.613 |
| F | 0.029 | 0.016 | 0.007 | 0.009 | 0.024 | G | 1.822 | 1.862 | 1.766 | 2.128 | 1.84 | 1.844 | 1.978 | 1.969 | 2.034 | 1.984 | 1.927 | 1.862 | 1.737 | 1.799 | 1.81 |
| G | 0.016 | 0.011 | 0.011 | 0.017 | 0.019 | 0.008 | H | 1.63 | 1.737 | 2.121 | 1.73 | 1.732 | 1.966 | 1.908 | 1.945 | 1.911 | 1.914 | 1.873 | 1.732 | 1.683 | 1.768 |
| H | 0.037 | 0.033 | 0.034 | 0.014 | 0.035 | 0.016 | 0.027 | I | 1.473 | 1.838 | 1.583 | 1.629 | 1.799 | 1.878 | 1.86 | 1.805 | 1.778 | 1.736 | 1.53 | 1.712 | 1.666 |
| I | 0.013 | 0.009 | 0.016 | 0.017 | 0.013 | 0.011 | 0.026 | 0.002 | K | 2.03 | 1.907 | 1.97 | 2.081 | 2.179 | 2.138 | 2.163 | 2.117 | 2.066 | 1.898 | 1.976 | 1.93 |
| K | 0.015 | 0.009 | 0.014 | 0.019 | 0.014 | 0.014 | 0.022 | 0.01 | 0.033 | L | 1.564 | 1.666 | 1.909 | 1.892 | 1.829 | 1.792 | 1.834 | 1.793 | 1.603 | 1.702 | 1.713 |
| L | 0.022 | 0.018 | 0.016 | 0.014 | 0.018 | 0.018 | 0.033 | 0.014 | 0.018 | 0.019 | M | 1.484 | 1.912 | 1.911 | 1.886 | 1.84 | 1.875 | 1.84 | 1.653 | 1.695 | 1.701 |
| M | 0.016 | 0.045 | 0.012 | 0.023 | 0.022 | 0.008 | 0.021 | 0.016 | 0.022 | 0.006 | 0.031 | N | 1.964 | 2.057 | 2.048 | 2.106 | 1.976 | 1.938 | 1.869 | 1.815 | 1.83 |
| N | 0.017 | 0.034 | 0.016 | 0.02 | 0.018 | 0.013 | 0.042 | 0.013 | 0.013 | 0.016 | 0.005 | 0.009 | P | 1.95 | 2.053 | 2.019 | 2.026 | 1.986 | 1.847 | 1.8 | 1.843 |
| P | 0.015 | 0.028 | 0.013 | 0.006 | 0.027 | 0.02 | 0.021 | 0.027 | 0.012 | 0.009 | 0.022 | 0.024 | 0.032 | Q | 1.947 | 2.008 | 2.004 | 2.009 | 1.867 | 1.824 | 1.891 |
| Q | 0.018 | 0.024 | 0.027 | 0.008 | 0.012 | 0.004 | 0.021 | 0.025 | 0.022 | 0.015 | 0.013 | 0.013 | 0.017 | 0.024 | R | 1.929 | 2.004 | 1.99 | 1.812 | 1.842 | 1.877 |
| R | 0.019 | 0.022 | 0.021 | 0.008 | 0.031 | 0.023 | 0.024 | 0.015 | 0.009 | 0.027 | 0.032 | 0.031 | 0.019 | 0.019 | 0.042 | S | 1.899 | 1.892 | 1.786 | 1.784 | 1.814 |
| S | 0.007 | 0.03 | 0.003 | 0.017 | 0.011 | 0.009 | 0.032 | 0.008 | 0.006 | 0.011 | 0.028 | 0.008 | 0.022 | 0.018 | 0.025 | 0.014 | T | 1.814 | 1.728 | 1.767 | 1.817 |
| T | 0.005 | 0.012 | 0.01 | 0.015 | 0.02 | 0.006 | 0.035 | 0.008 | 0.004 | 0.016 | 0.02 | 0.022 | 0.022 | 0.006 | 0.017 | 0.013 | 0.008 | V | 1.503 | 1.698 | 1.691 |
| V | 0.008 | 0.006 | 0.014 | 0.014 | 0.016 | 0.003 | 0.035 | 0.011 | 0.018 | 0.012 | 0.015 | 0.018 | 0.017 | 0.017 | 0.02 | 0.002 | 0.005 | 0.013 | W | 1.383 | 1.591 |
| W | 0.031 | 0.038 | 0.023 | 0.016 | 0.032 | 0.019 | 0.024 | 0.031 | 0.004 | 0.014 | 0.041 | 0.023 | 0.018 | 0.026 | 0.012 | 0.012 | 0.017 | 0.012 | 0.072 | Y | 1.623 |
| Y | 0.01 | 0.041 | 0.013 | 0.012 | 0.017 | 0.015 | 0.019 | 0.014 | 0.005 | 0.012 | 0.017 | 0.021 | 0.026 | 0.021 | 0.025 | 0.012 | 0.026 | 0.007 | 0.024 | 0.016 |  |
